# The Interplay of Microtubules with Mitochondria–ER Contact Sites (MERCs) in Glioblastoma

**DOI:** 10.3390/biom12040567

**Published:** 2022-04-12

**Authors:** Francesca Grespi, Caterina Vianello, Stefano Cagnin, Marta Giacomello, Agnese De Mario

**Affiliations:** 1 Department of Biology, University of Padua, Via Ugo Bassi 58b, 35100 Padua, Italy; francesca.grespi@gmail.com (F.G.); caterina.vianello@unipd.it (C.V.); stefano.cagnin@unipd.it (S.C.); 2 CRIBI Biotechnology Center, University of Padua, Via Ugo Bassi 58b, 35100 Padua, Italy; 3 CIR-Myo Myology Center, University of Padua, Via Ugo Bassi 58b, 35100 Padua, Italy; 4 Department of Biomedical Sciences, University of Padua, Via Ugo Bassi 58b, 35100 Padua, Italy

**Keywords:** glioblastoma, glioblastoma invasion, glioma, MAMs, MERCs, microtubules, cytoskeleton, mitochondrial dynamics, gene expression

## Abstract

Gliomas are heterogeneous neoplasms, classified into grade I to IV according to their malignancy and the presence of specific histological/molecular hallmarks. The higher grade of glioma is known as glioblastoma (GB). Although progress has been made in surgical and radiation treatments, its clinical outcome is still unfavorable. The invasive properties of GB cells and glioma aggressiveness are linked to the reshaping of the cytoskeleton. Recent works suggest that the different susceptibility of GB cells to antitumor immune response is also associated with the extent and function of mitochondria–ER contact sites (MERCs). The presence of MERCs alterations could also explain the mitochondrial defects observed in GB models, including abnormalities of energy metabolism and disruption of apoptotic and calcium signaling. Based on this evidence, the question arises as to whether a MERCs–cytoskeleton crosstalk exists, and whether GB progression is linked to an altered cytoskeleton–MERCs interaction. To address this possibility, in this review we performed a meta-analysis to compare grade I and grade IV GB patients. From this preliminary analysis, we found that GB samples (grade IV) are characterized by altered expression of cytoskeletal and MERCs related genes. Among them, the cytoskeleton-associated protein 4 (CKAP4 or CLIMP-63) appears particularly interesting as it encodes a MERCs protein controlling the ER anchoring to microtubules (MTs). Although further in-depth analyses remain necessary, this perspective review may provide new hints to better understand GB molecular etiopathogenesis, by suggesting that cytoskeletal and MERCs alterations cooperate to exacerbate the cellular phenotype of high-grade GB and that MERCs players can be exploited as novel biomarkers/targets to enhance the current therapy for GB.

## 1. Introduction

Glioblastoma (GB), also known as glioblastoma multiforme, is the most aggressive type of astrocytoma. GB derives both from glial and glioma stem cells and affects glia and astrocytes [1]. Although the current therapeutic strategy accounts for complementary approaches, as surgical resection, radiotherapy, chemotherapy and antiangiogenesis agents, the prognosis of GB patients is still poor [2].

Based on well-defined genome-wide gene expression changes, somatic mutations and copy number changes, GB is commonly divided into four subtypes: classical, proneural, mesenchymal, and neural [3,4]. The classical subtype (97% of tumors) is characterized by an altered expression of the epidermal growth factor receptor (EGFR) while the proneural subtype by alterations in the genes coding for the tumor protein p53 (TP53), platelet-derived growth factor receptor (PDGFRA), isocitrate dehydrogenase-1 (IDH1) [5]. The mesenchymal subtype is due mostly to mutations in the neurofibromin 1 (NF1) gene, while a low percentage of the cases are linked to EGFR gene changes. The mesenchymal subtype is also enriched in the expression of astrocytes and microglia markers. The neural subtype is enriched in astrocytic and oligodendrocytic markers such as Oligodendrocyte transcription factor 1 (OLIG1), 3-O-methyl-[3H]-glucose (OMG), proteolipid protein 1 (PLP1), Tenascin-R (TNR), Glial fibrillary acidic protein (GFAP), Excitatory amino acid transporter 1 (SLC1A3), astrocyte-specific glutamate transporter (GLAST), Megalencephalic leukoencephalopathy with subcortical cysts protein-1 (MLC1), SRY-Box Transcription Factor (SOX) 4, SOX11, and Doublecortin (DCX) [6,7].

Most of the GB cases are idiopathic: only a minority of patients (~5%) harbor critical germline alterations, while 20% have a strong family history of cancer [8]. GBs arise mainly in the supratentorial cerebral hemispheres, with variable clinical presentations. GB symptoms include persistent weakness, numbness, loss of vision or alteration of language; those located in the frontal lobe, temporal lobe, or corpus callosum are associated with, executive dysfunctions, mood disorders, fatigue and mild memory disorders [9].

Gliomas are classified by the WHO (World Health Organization Histological Classification of Tumors) into different grades based on the severity of the disease—grade IV being the most aggressive—with a mean survival of 15 months from the diagnosis [10]. The effectiveness of therapeutic treatments is hampered by many factors like heterogeneous molecular and genetic backgrounds of the different tumors and of the various cells within the same tumor [2,10,11]. Despite many studies aimed to investigate the molecular mechanisms underlying GB, its cellular origin remains enigmatic. It has been hypothesized that GB raises from neuroectodermal stem cells: endowed of high proliferative potential, the latter display strong migratory abilities and can undergo differentiation into GB cells [12]. However, the cells that populate GB are not homogeneous: the tumor mass contains endothelial cells, stem cells, astrocytes, progenitor cells and immune cells.

During GB progression the blood brain barrier (BBB) is compromised, allowing entry of immune cells and neuroinflammation due to chemoattraction and activation of glial cells. Microglial cells produce high levels of proinflammatory molecules, such as nitric oxide (NO) and tumor necrosis factor alpha (TNF-α), further enhancing BBB breakdown by disruption of the astrocyte–BBB junctions (as comprehensively described in [13,14,15]. The heterogeneous nature of GB cells also underlies their distinct susceptibility to cytotoxic T lymphocytes, which are crucial for immune defense. Accordingly, Bassoy and co-authors found that glioma stem cells were more readily attacked and killed in vitro and in vivo by cytotoxic T lymphocytes than their differentiated counterpart [16]. Here the authors show that the interaction among the Endoplasmic Reticulum (ER) and mitochondria drives the expression of surface glycans in glioma stem-like cells and thereby dictates their response to killer lymphocytes [17].

GB rarely metastasize outside the central nervous system (CNS). However, it can be highly invasive within the brain parenchyma, a feature that severely limits the efficacy of surgery and radiotherapy. Invasion of GB cells into the brain reflects the dynamic interplay between cell-to-cell adhesion, remodeling of the extracellular matrix and cell motility. Specifically, during epithelial-mesenchymal transition (EMT), cancer cells enhance migratory and invasive capabilities through the reorganization of the cytoskeleton and the formation of membrane protrusions [18]. Treatments targeting Rho GTPases, that modulate cell migration through downstream rearrangement of the actin cytoskeleton, have been proposed as a novel approach for GB treatment [19,20].

Molecularly, GB have been associated with alterations in cell cycle regulatory genes such as p16, cyclin-dependent kinase (cdk) 4, cdk6, cyclin D1 and retinoblastoma protein, as well as of molecules belonging to tumor activation pathways like receptor tyrosine kinases responsible for activation of the Phosphoinositide 3-kinases (PI3K), Protein kinase B, (Akt), mammalian target of rapamycin (mTOR) and mitogen-activated protein kinase (MAPK) axes [21]. As in other solid tumors, dysfunction in mitochondria physiology has been reported and is considered a key driver of GB progression. Abnormalities of the cell bioenergetics, changes in mitochondria membrane potential regulation, disruption of apoptotic signaling pathways, mitochondrial swelling with partial or total breakdown of mitochondrial cristae (cristolysis) and deranged fusion and fission processes have been observed [22,23]. These ultrastructural changes are consistent with the alterations of mitochondrial bioenergetics, pointing to a critical derangement of oxidative phosphorylation (OXPHOS) in GB cells [23].

These pathways are controlled both by the interaction among ER and mitochondria at the mitochondria–Endoplasmic Reticulum contact sites (MERCs [24]) and by cytoskeletal remodeling, thus suggesting that these two factors cooperate towards the development of GB. In this review we discuss this possibility, that is: the role of microtubule rearrangement and its interplay with MERCs in the progression of GB.

## 2. Cytoskeleton and Glial Cells: A Focus on Microtubule and Microfilaments

The cytoskeleton is formed by three different types of dynamic filaments that determine the cell structural organization: microtubules (heterodimers of α- and β-tubulins), intermediate filaments (neurofilaments or glial filaments) and microfilaments (two strands of actin subunits). In this review we will focus mainly on microtubules (MTs). Accounting for 20% of total protein in the brain, microtubules are involved in important cell functions: mitotic spindle formation, ensuring proper chromosome segregation and cell division, maintenance of the cellular architecture and structure, intracellular organelles transport and signal propagation. MTs interact with diverse organelles, including the endoplasmic reticulum (ER), Golgi apparatus, lysosomes and mitochondria [25,26,27]. MTs assemble from dimers of α- and β-tubulin: the latter align head-to-tail to form protofilaments, which in turn associate laterally and form tubes. The extremity where α-tubulin is exposed (termed the minus end) grows slowly in vitro, whereas the opposite end (termed the plus end) grows rapidly [28]. Assembly (polymerization) and disassembly (depolymerization) of microtubules is driven by hydrolysis of guanosine triphosphate (GTP) on the β-tubulin monomer. Polymerization is typically initiated from a pool of GTP-loaded tubulin subunits [29]. GTP hydrolysis occurs shortly after incorporation of tubulin subunits and induces a conformational change of protofilaments: from a slightly curved tubulin-GTP bound rod to a more profoundly curved tubulin-guanosine diphosphate (GDP) structure [29]. Growing microtubule sheets maintain a “cap” of tubulin-GTP subunits whose loss results in rapid depolymerization. The microtubule network is controlled by many proteins, such as microtubule associated proteins (MAP), the stabilizing proteins Microtubule-associated protein RP/EB family member 1 and Cytoplasmic linker protein CLIP-170, as well as by microtubule-destabilizing or depolymerizing proteins, like spastin and katanin, the depolymerizing motor protein kinesin and the αβ-tubulin dimer-binding protein stathmin [30]. MTs interact also with regulators of the cell cycle and apoptosis, including the tumor suppressor protein p53, Bcl-2 and survivin and with the intracellular transport proteins kinesins and dyneins [31]. Dyneins transport various cell cargo by crawling along cytoskeletal MTs towards their minus-end. Kinesin family members (KIF) are instead a group of proteins playing a role in cytoskeleton organization: KIF move along microtubule filaments after hydrolysis of ATP and are responsible for their assembly into parallel arrays [32]. Post-translational modifications (tyrosination, glutamylation, and acetylation) also control their polymerization, the functions of the microtubule network (e.g., chromosome segregation, spindle formation, transport of organelles, including mitochondria [33,34] and the affinity for Microtubule-Associated Proteins (MAPs) [35]. MAPs are key regulator of MTs dynamics, as they can (de) stabilize protofilaments, guide their growth towards specific cellular domains, mediate their interaction with other proteins. Tubulin is also subjected to palmytoilation (that is, protein fatty acid acylation) [36]. This covalent attachment of lipids allows anchoring of organelles membranes to the tubulin filaments and their positioning along the MTs.

The organization of microtubules differs among cell types: in neurons, the MTs network determines cell polarity to support the growth and structure of axon and dendrites [37]. The glial environment constantly influences the axonal cytoskeleton, providing an example of the dynamic nature of the neuronal cytoskeleton and of the interplay among different cell types in the brain [38]. Indeed, radial cells contribute to the overall structural and functional organization of the central nervous system via microtubule dependent processes. This is demonstrated by the fact that lissencephaly (a clinical condition in which deranged cortical development affects the surface of the brain, due to the failure of neuronal migration) is associated with altered biogenesis of radial glia microtubules [39]. The microtubule cytoskeleton controls the morphology and polarization of the glial cells. It also provides a dynamic platform for the development and extension of membrane processes at the basis of inter-cellular communication and migration. While on one hand these evidences point to the central role of MTs for glial cells function, they also support the possibility that altered microtubule dynamics contribute to the development of human disorders characterized by altered cell motility [40]. It is important to briefly mention the role of actin filaments in regulating brain cells physiology. In general, actin filaments are crucial for various processes such as proliferation, migration, cell morphogenesis and apoptosis (for a more detailed and comprehensive overview of actin dynamics and function we refer the reader to [41,42,43,44]. In the case of microglia, actin plays a fundamental role by shaping its physiology and motility [41,45,46,47] -actin monomers assemble into actin filaments (F-actin) that form lamellipodia and filopodia which are responsible of cell movement.

Cytoskeleton dynamics are also shaped by other intermediate filaments proteins, which are expressed in a tissue-specific manner, in contrast to actin microfilaments and microtubules. Among these, vimentin is particularly important, being fundamentally expressed in radial glia and immature astrocytes during development. Vimentin filaments are characterized by higher elasticity than actin and microtubules, a property which is typical also of desmin, the major intermediate filament protein of muscle [48]. Vimentin importance is highlighted by the fact that vimentin intermediate filaments and actin cooperate to redefine the cell cortex. Further, Vimentin is a key component of migrasomes, recently discovered vesicles located within retraction fibers that control the cell migration speed [49]. Migration does not depend on the function of a single protein class. Rather, it depends on the interplay among different types of intermediate filaments, as in the case of epithelial cells whose migration requires the interaction between vimentin and keratin intermediate filaments [50]. Finally, intermediate filaments proteins include lamins, which play an important role in maintaining the shape of the nuclei of all cells, as well as in the organization of the cytoskeleton, mechanotransduction, and cell motility [51].

## 3. Cytoskeletal Proteins: Key Players of Glioblastoma Cell Invasive Properties

Recent reports indicate a role for MTs in GB progression and invasion. As migration and infiltration of GB cells is governed by reshaping of the cytoskeleton, it is not surprising that the composition and organization of the cytoskeleton in GB cells differs from that of healthy brain cells. Besides the altered expression of tubulin isoforms [52,53], many findings link GB to altered microtubule dynamics, especially those mediated by microtubule associated proteins (MAPs). Microarray analyses and immunohistochemical studies have found that GB tissues express an astrocytoma-specific splice variant (MAP2e) of the neuronal MAP2, particularly in the cells at the invasive front of the tumor [54]. Another MAP overexpressed in GB cells is the receptor for hyaluronic acid mediated motility (RHAMM)/intracellular hyaluronic acid binding protein (IHABP) that exhibits several splice variants, some of which are specific to GB [55].

Invading Cells are also enriched in dynein, in dynein-associated and actin-associated proteins, substantially contributing to the subcellular processes underlying their proliferative and migration abilities [56]. Notably, advillin (AVIL), a member of the villin/gelsolin family that regulates actin filament reorganization, also contributes to GB tumorigenesis [57]. Xie and colleagues found that AVIL is overexpressed in GB cells (including GB stem cells), and that its overexpression promotes cell movement, GB proliferation and migration. The latter are fostered in GB also by overexpression of formin (FMN1), that controls polymerization of actin cables, e.g., linear cytoskeletal structures that assemble at nascent adherens junctions and drive intercellular adhesion [58,59].

Another cytoskeletal protein linked to GB is Nestin, a class IV intermediate filament protein that is considered as a putative marker of neural progenitor stem cells in the central nervous system. Not only Nestin expression is significantly overexpressed in GB, compared with other types of glioma, but its deletion in tumor cells from GB patients derived xenografts caused cell cycle blockage, finally bringing to tumor cells death. This effect is due to the physical association of Nestin with β II-tubulin: indeed, deletion of either Nestin or βII-tubulin disrupted spindle morphology in GB cells [60].

Higher expression levels of lamin A/C have been also linked with a reduced overall survival of patients affected by glioblastoma multiforme, as well as with the enrichment of cancer-related pathways related to cell migration and adhesion [61]. Recent analyses also highlight that some keratin forms (KRT33B, KRT75) can be used as prognostic biomarkers for early detection of low-grade gliomas, and others (KRT75) can be used to reveal transformation tumors with higher probability to switch from low-grade gliomas to GB [62].

Overall, these findings suggest the existence of a relationship between the invasive properties of GB cells and the derangement of cytoskeleton network and functions.

## 4. Mitochondria Dynamics and Glial Cells

Proper MTs organization is essential also for the movement of mitochondria, whose dysfunctions have been also associated with the infiltrative and proliferative capacity of glioma cells [18,63]. Anterograde and retrograde transport of mitochondria along MTs are mediated by plus end-directed kinesin-1/KIF5 and minus-end-directed dynein motor proteins respectively [64]. The interaction between mitochondria and kinesin/dynein depends on the formation of a complex between the outer mitochondria membrane (OMM), the mitochondrial rho GTPase Miro 1 (Miro1) and TRAK1/2 (also known as Milton) [65]. Miro-1 binds the cytoplasmic adaptor protein Milton and the kinesin heavy chain through its cytoplasmic domain, thereby connecting mitochondria to microtubules [66]. The movement of the organelles is tightly coordinated with changes in their morphology [26]. Their shape and assembly into a network result from a net balance of fusion–fission processes of the mitochondria membranes. These processes are regulated by a family of “mitochondria-shaping proteins”: e.g., in mammals, fission is controlled by the concerted action of the cytosolic Dynamin-1-like protein (DRP-1) [67] and of proteins that recruit Drp1 at the surface of mitochondria like human Mitochondrial fission 1 protein (hFis1), Mitochondrial elongation factor 2 (Mid49), Mitochondrial fission factors (MFFs). Among the protein that control fusion, the most studied are Optic Atrophy 1 (Opa1) and Mitofusins (Mfn; for a comprehensive view of mitochondrial dynamics, see [68]). As mentioned before, several defects in mitochondrial morphology, positioning and functionality have been described in brain cancer cells [69] and in other cancer cells [70].

The subcellular distribution of mitochondria has been correlated with the migration and invasion capabilities of GB cells [71]. It has been shown that PI3K antagonists trigger the transport of energetically active, elongated mitochondria to the cortical cytoskeleton of tumor cells. The repositioned mitochondria apparently speed up lamellipodia dynamics, induce faster turnover of focal adhesion complexes, increase speed and width of cell migration, ultimately leading to higher rates of GB cell invasion [52,72,73]. Mitochondria support GB invasiveness not only though ATP supply, but also by controlling the expression of surface glycans in glioma stem-like cells [16]. Indeed, although the exact mechanism has not been completely clarified, Bassoy and colleagues showed that ER–mitochondria interactions, rather than mitochondria per se, determine GSC sialylated glycan surface expression. The crosstalk of mitochondria and ER fosters the expression of the sialylated glycans, that are subsequently exposed to the cell surface and contribute to cell adhesion [74].

Mitochondria–ER contact sites (MERCs), the sites of contacts among ER and mitochondria, participate to different cell functions and pathologies [24,75,76]. The environment generated by the MERCs represents a privileged site for non-vesicular lipid trafficking between these organelles and facilitates transport of lipids via carrier proteins [77]. Several key enzymes, located in MAMs, mediate phospholipid synthesis, such as phosphatidylserine synthase-1/2 (PSS1/2) and phosphatidylethanolamine N-methyltransferase 2 (PEMT2), [78], while oxysterol-binding protein (OSBP)-related protein 5 and 8 (ORP5/ORP8) have been recently reported to locate at MERCs where they mediate the transport of phospholipids [79]. In addition, key regulators of triacylglycerol synthesis and steroidogenesis, such as acyl-CoA/diacylglycerol acyltransferase 2 (DGAT2) [80], the steroidogenic acute regulatory protein (StAR) [81], the long-chain-fatty-acid-CoA ligase 4 (FACL4) [82] are enriched at MERCs. The cholesterol acyltransferase-1 (ACAT1/SOAT1), an enzyme that catalyzes the generation of cholesterol esters, is also enriched in MERCs. The contribution of MERCs to lipid homeostasis appears fundamental also for the etiology of cancer: the amount and proportion of the various types of lipids available within cells dictate their membrane composition, fluidity and stiffness, ultimately controlling their adhesion ability. Although a detailed overview is beyond the scope of this review (please refer to [24,76] for a more comprehensive description), we want to highlight here another MERCs function that is a decision-making node in the balance between cell survival and death: its contribution to the regulation of Ca^2+^ homeostasis [83,84,85]. Thanks to the tight tether between the ER membranes and mitochondria, Ca^2+^ can be rapidly transferred through the formation of MERCs-resident microdomains that overcome the low Ca^2+^ affinity of the mitochondrial Ca^2+^ uniporter (MCU) [83,86]. The IP3R is physically linked to the OMM voltage-dependent anion channel 1 (VDAC1) by means of the molecular chaperone glucose-regulated protein 75 (Grp75) [87]. Moreover, the sarco/endoplasmic reticulum Ca^2+^ ATPase (SERCA) pump localized to the ER membrane is regulated by several proteins residing at MERCs, thereby modulating Ca^2+^ kinetics [88].

As reported above, mammalian cells proteins involved in the anterograde and retrograde movement of organelles are controlled by local Ca^2+^ rises. For example, the binding of Ca^2+^ to the EF hands domains of the OMM Rho-GTPase protein Miro1 drives its conformational change and decoupling from microtubule motors (specifically, kinesin and dynein), thereby controlling mitochondrial motility [89]. Not only do cytosolic Ca^2+^ levels control mitochondrial transport, but they also control changes in the concentration of mitochondrial matrix Ca^2+^: of note, it has been demonstrated that Miro1 regulates intramitochondrial Ca^2+^ levels. Although Miro1 has been shown to localize at MERCs, where it contributes to lipids exchange and biosynthesis [90,91], and although it has been reported to interact/be regulated with/by MERCs-associated proteins (e.g., Mitofusin, Parkin, Pink1 [92,93]), how it functions within MERCs relates with its extra-MERCs role, which has not been completely clarified.

Finally, while regulated Ca^2+^ transport at MAMs favors bioenergetics, it is well established that massive and prolonged mitochondrial Ca^2+^ load promotes increased reactive oxygen species production and may trigger the opening of the mitochondrial permeability transition pore, eventually leading to apoptosis [94]. As a result of the consequent OMM permeabilization, caspase-activating and proapoptotic factors, including cytochrome c, are released into the cytoplasm, exacerbating Ca^2+^ release from the IP3R and avoiding the Ca^2+^-dependent inhibition of the receptor itself [95].

All these findings suggest a specialized role of MERCs in glial cells by influencing the mitochondria and Ca^2+^-dependent processes that support their normal physiology.

## 5. MERCs in Glioblastoma

Considering the role of MERCs in the maintenance of cell homeostasis, it is not surprising that some experimental reports highlighted their contribution to the etiology of different types of tumors, including GB.

A first link between MAMs and cancer has been shown in case of the protein promyelocytic leukemia (PML), a nuclear tumor suppressor protein, which has been located at MERCs. Here, PML modulates the IP3R-Akt axis trough the regulation of Ca^2+^ transfer from the ER to the mitochondria and ultimately induces pro-apoptotic signaling [96]. Notably, the Akt pathway has been also connected with the increased tumorigenicity, stem cell-ness, and invasiveness of GB cells [97]. This could strengthen the MERCs-dependent effect on glycan expression that has been associated with enhanced migration abilities and diverse susceptibility to cytotoxic lymphocyte-mediated killing of GB cells [16]. Interestingly, studies on MERCs ultrastructure in surgical specimens obtained from human astrocytic neoplasms revealed a striking difference among well-differentiated glioma cells and poorly differentiated glioma like-stem cells. The latter appeared almost deprived of MERCs [98]. Although this finding is interesting, it remains unclear whether this is a typical feature of stem cells and of stem cell-like cells, which could be MERCs contribution to glioma stem cells physiology. Notably, glioma stem cells appear less glycolytic than their differentiated counterparts and rely mainly on oxidative phosphorylation [99]. At variance, differentiated GB cells were reported as “MERCs-enriched”, with MERCs driving mitochondria functionality and sustaining their metabolic demands [100].

Another protein important in the context of tumors is the cytoskeleton-binding protein trichoplein/mitostatin (TpM), which binds the major intermediate filaments of epithelial cells. TpM acts as a negative modulator of ER–mitochondria juxtaposition, inhibiting apoptosis induced by Ca^2+^-dependent stimuli through interaction with MFN2 [94]. Interestingly, TpM is involved in the formation of the primary cilium, a dynamic protrusion present in virtually every type of cell that senses extracellular signals and coordinates the subcellular responses with the progression of cell cycle, proliferation, and differentiation. This is particularly interesting in the context of GB, since aberrant ciliogenesis has been proposed as an early event of GB etiology [101,102]. The potential contribution of TpM to carcinogenesis is corroborated by findings that regards other types of solid tumors. As an example, it has been demonstrated that TpM inhibits cell migration, invasion, and tumorigenicity of prostate cancer cells, and that it is down-regulated in advanced stages of human prostate cancers [95]. All these data suggest that TpM can act as a tumor suppressor: however, it has not been clarified whether these findings are due to altered TpM function at MERCs or to variations of its extra-MERCs physiological roles.

## 6. MERCs and Microtubules Crosstalk in Glioblastoma

In the previous paragraphs, we summarized the published findings on the potential contribution of MERCs and cytoskeletal dynamics to GB etiology. Considering the role of the latter in the maintenance of the cell bioenergetics, signaling, and migration abilities, the question rises as to whether and how MERCs and MTs contribute and coordinate their activities during GB growth and diffusion into different brain regions. The finding that the interaction between tubulin and the MERCs–resident protein VDAC1 actively controls the cell metabolism in muscle and in cell models of neuroblastoma and breast cancer hints at this possibility [103]. Data from different cell models support the idea that tubulin can control mitochondrial respiration by diminishing the VDAC-mediated permeability of the OMM to ATP, ADP, and other metabolic substrates [104,105]. It is tempting to speculate that this could be a mechanism to shape mitochondrial respiration and support the metabolic changes that characterize GB [106]. Interestingly, VDAC1 binding to tubulin dimers and the consequent blockage of its activity is controlled by phosphorylation either by glycogen synthase kinase-3β or by cyclic AMP-dependent protein kinase (PKA), whose expression differs in glioma cells with respect to normal tissue [107,108,109]. Theoretically, altered levels of these kinases could influence the levels of phosphorylated VDAC1 and in turn influence its tubulin binding, a possibility that has not yet been demonstrated in the context of GB.

In addition, several recent studies have utilized high-throughput genomic, epigenomic, and transcriptomic approaches for detailed molecular characterization of GB. A recent proteomic study found that cells derived from GB patients upregulate the expression not only of cytoskeletal (vimentin, α- and β-tubulin, β-actin, and glial fibrillary acidic protein), but also of mitochondrial (aldehyde dehydrogenase and manganese superoxide dismutase) and MERCs/ER-resident chaperone proteins (GRP-75, GRP-57, and HSP27) [109].

To provide details on the crosstalk among MERCs and the cytoskeleton in glial cells tumors, we took advantage of published genome-wide gene expression analyses to evaluate alterations occurring during GB progression. We identified differentially expressed genes (DEGs) in GB considering two GEO datasets that are unique because they were obtained upon analysis of human GB biopsies (GSE147352 and GSE79878) [110]. These datasets include 93 glioblastoma, 18 glioma, and 15 normal samples studied though two different techniques: microarray and RNA sequencing.

DESeq normalized read counts were used to identify differentially expressed genes using the AltAnalyze software [111]. Genes were considered as differentially expressed whenever their fold change would be > 2 and *p*-value < 0.05.

**Figure 1:**
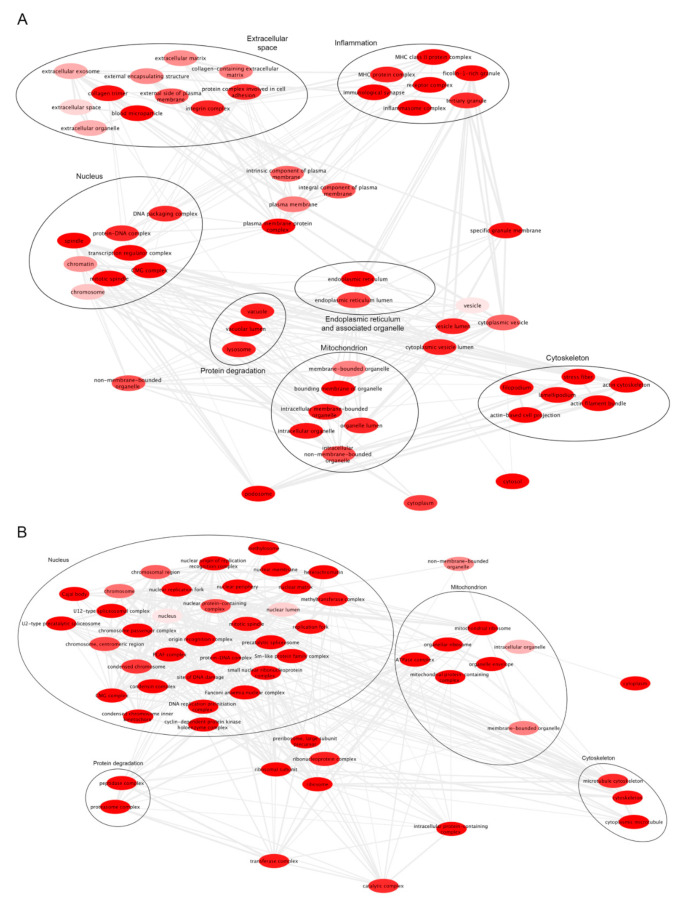
Enriched GO terms. (**A**). GO enrichment from upregulated genes in GB from GSE147352 dataset. (**B**). GO enrichment from upregulated genes in GB grade IV from GSE79878 dataset. The bubble color indicates the FDR for the GO term (bright red lower FDR). Highly similar GO terms are linked by edges in the graph.

In case of microarray gene expression analysis, we excluded lincRNAs from the analysis before normalizing data because they are lower expressed than coding RNAs, as we did in [112]. Raw data with gene expression information for coding genes were quantile normalized using R, and the expression of each gene was calculated as the average of multiple probes for the same gene with an intensity fluorescence significantly above the background (using the filter gIsPosAndSignif in the Agilent microarray quantification files [113]). DEGs were identified using TMev_4.8.1 software (Quantitative Biomedical Research Center, Harvard T.H. Chan School of Public Health, Boston, USA) [114] (www.mev.tm4.org, accessed on 5 April 2022). Genes were considered altered when fold change was > 1.5 and *p*-value < 0.05. DEGs (Supplementary Table S1) were categorized according to Gene Ontology (GO) using the Panther 17.0 database [115] (www.pantherdb.org, accessed on 17 January 2022) (Supplementary Table S2) and GO terms were reduced using the REVIGO tool with default parameters [116]. Downregulated genes in GB were involved in neuronal functions as the formation of neuron projection or in the excitatory of GABAergic signal (Supplementary Figure S1 and Table S2). The list of upregulated genes in GB was also enriched of molecules controlling cytoskeletal and mitochondrial functions (Figure 1). By analyzing RNAseq data (GSE147352) (microarray analyses are based on the comparison between gliomas grade I and GB grade IV), we highlighted that genes associated with ER are also expressed (Figure 1A).

**Figure 2:**
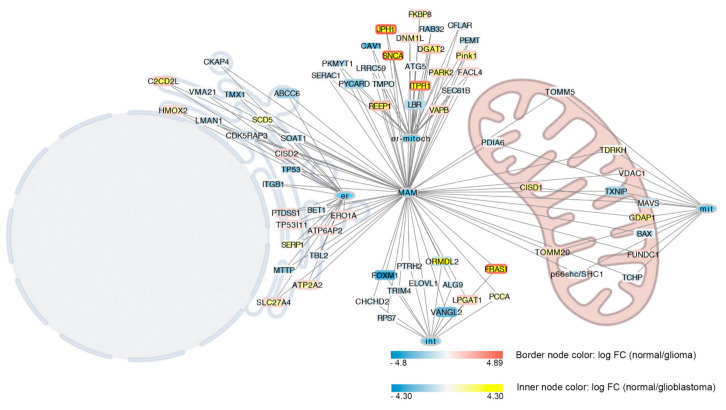
Differentially expressed genes involved in MERC interactions. Genes associated to MERC interactions are represented according to subcellular localization of coded proteins. Proteins specifically localized in the endoplasmic reticulum are associated with the node labelled as er, those associated with mitochondria are associated with the node labelled with mit, and those localized both in ER on in the mitochondria are associated with the node labelled with er–mitoch. Proteins involved in MERCs according to [104] not specifically associated with ER or mitochondria were linked to a node labelled with int. All nodes are linked to the MERCs node because all are involved in the formation of MERCs. MERCs, ER, mitochondria, and ER–mitochondria nodes are colored with light blue while other nodes are colored according to the log FC expression of control versus high grade glioblastoma. Differently node border is colored according to the log FC expression of control versus glioma. Blue indicates highly expressed in the tumor, yellow or red in the control, and white indicates non-differences.

We then evaluated alterations in genes involved in the synthesis of MERCs proteins (Supplementary Table S3). Using normal samples as control, we found that 37% (62 of 169) of genes coding for MERCs proteins were altered in GB (Supplementary Table S3), and 22% (38 of 169) were altered in glioma tumors (Supplementary Table S3 and Figure 2). Interestingly, most of the genes coding for proteins controlling MERCs formation were also differentially expressed in glioblastoma samples. Genes most downregulated in glioblastoma are also highly expressed in glioma (JPH1, SNCA, ITPR1, and FRAS1). On the other hand, those highly expressed in glioblastoma (CAV1, TP53, TMX1, MTTP, RAB32, ITGB1, PEMT, SOAT1, BAX, PKMYT1) are not altered in glioma (Figure 2 and Supplementary Table S3). Genes involved in MAMs formation whose expression was altered only in glioma samples were FUNDC1, FACL4, VDAC1, ATP6AP2, CISD2, ERO1A, TP53I11, MAVS, and CDK5RAP3, while those altered only in GB and not in gliomas were TDRKH, SOAT1, LMAN1, TRIM4, CAV1, BAX, ITGB1, SEC61B, PDIA6, CFLAR, RAB32, MTTP, TMPO, ELOVL1, PKMYT1, p66shc/SHC1, PEMT, LRRC59, BET1, TBL2, ALG9, TOMM5, SERAC1, TCHP, VMA21, RPS7, PTRH2, ATG5, CHCHD2, SCD5, SERP1, PCCA, and CKAP4 (Supplementary Table S3).

Among the identified DEGs, CKAP4, Cytoskeleton-associated protein 4, appears particularly interesting. The corresponding protein is also known as Cytoskeleton-Linking Membrane Protein 63 (CLIMP-63) or p63, because it is a reversibly palmitoylated protein located in ER which stabilizes the ER structure and mediates the interaction of this organelle with MTs [117]. Notably, CKAP4 plays a role in maintaining mitochondrial functions through binding to VDAC2 at ER–mitochondria contact sites [118]. It also forms a complex with PI3K upon the binding of Dickkopf WNT Signaling Pathway Inhibitor 1 (DKK1), leading to the activation of Akt, which has been implied in glial cell tumors, as specified above. Both DKK1 and CKAP4 are frequently expressed in pancreatic and lung tumors, and their simultaneous expression is inversely correlated with cancer prognosis [119].

Another important interactor protein of CKAP4 is VIMP, Valosin-containing protein-interacting membrane protein (VIMP): together, they link the ER with microtubules [120]. Indeed, VIMP contributes to the organization of the ER acting as a scaffold for CLIMP-63 and polymerized MTs. Interestingly, we identified VIMP among the cytoskeletal genes upregulated in GB, suggesting the possibility that VIMP and CKAP4 could integrate the mitochondria–ER and cytoskeleton dynamics and functions, potentially contributing to the progression of GB.

## 7. Conclusions

GB is the most malignant glial tumor in the brain, with a devastating prognosis because of its complex biological behaviors and limited therapeutical strategies. While abnormalities in the expression/function of cytoskeletal proteins and in microtubules associated proteins in GB have been already reported [54,121], the potential contribution of MERCs in GB has been suggested only recently [65]. Recent advances in the knowledge of mitochondrial biology, with consequent implications in cancer, highlight the existence of interactions among cytoskeletal and OMM proteins implied in the control of mitochondrial functions and consequently of tissue-specific metabolic outcomes [65,121].

Here we propose for the first time the existence of a crosstalk among MTs and MERCs in the context of glial cells cancer. By identifying genes which are differentially expressed in normal samples with respect to GB, or in different GB grades (grade I versus grade IV), we found that about 37% of the known genes coding for MERCs proteins are abnormally expressed. This finding goes along with the altered expression of genes coding for cytoskeletal components or regulators. Both MERCs and microtubule dynamics are strong determinants of mitochondrial function: theoretically, their defects could also account for the metabolic hallmarks that characterize GB. Of course, future studies are needed to analyze in detail the MERCs–microtubules axis in GB, as our preliminary data rely on gene expression levels. Connecting them with changes in the function of MERCs and microtubules proteins requires further experimental analyses, guaranteeing new knowledge on the etiology of this severe brain tumor.

## Data Availability

We have used the following GEO datasets: GSE147352 and GSE79878.

## Supplementary Materials

The following supporting information can be downloaded at: https://www.mdpi.com/article/10.3390/biom12040567/s1, Figure S1. Tree Map. Results describes a summarization of GO terms of downregulated genes in GB. Analysis was performed using REVIGO application with default parameters. Each rectangle is a single cluster representative that are joined into ‘superclusters’ of related terms (same color). Table S1. Differentially expressed genes in GB and Glioma. Table S2. Differentially expressed genes categorized according to Gene Ontology. Table S3. MERCs genes altered in GB and in Glioma.
