# Supplementary figures and images for: The Interplay of Microtubules with Mitochondria–ER Contact Sites (MERCs) in Glioblastoma
Source: Biomolecules. 2022 Apr 12;12(4):567. doi: 10.3390/biom12040567 (PMC9030160; doi:10.3390/biom12040567)

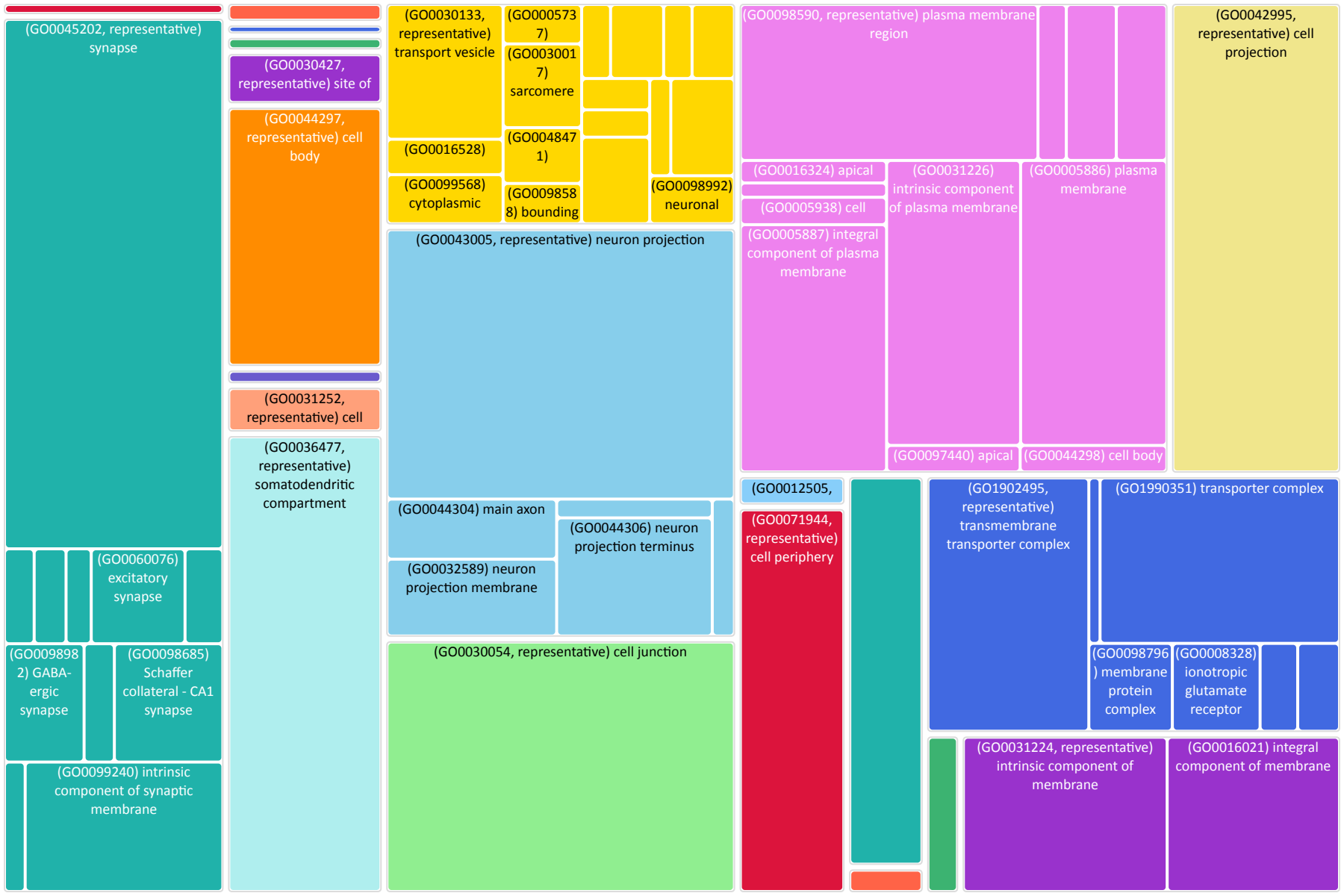

Supplement: Supplementary file 1 [file biomolecules-12-00567-s001.zip › Figure-S1.pdf]
